# Evaluating DeepResearch and DeepThink in anterior cruciate ligament surgery patient education: ChatGPT‐4o excels in comprehensiveness, DeepSeek R1 leads in clarity and readability of orthopaedic information

**DOI:** 10.1002/ksa.12711

**Published:** 2025-06-01

**Authors:** Onur Gültekin, Jumpei Inoue, Baris Yilmaz, Mehmet Halis Cerci, Bekir Eray Kilinc, Hüsnü Yilmaz, Robert Prill, Mahmut Enes Kayaalp

**Affiliations:** ^1^ Department of Orthopaedics and Traumatology, Istanbul Fatih Sultan Mehmet Training and Research Hospital University of Health Sciences Istanbul Turkey; ^2^ Department of Orthopaedic Surgery Nagoya Tokushukai General Hospital Kasugai Aichi Japan; ^3^ Department of Orthopedics and Traumatology Memorial Sisli Hospital Istanbul Turkey; ^4^ Department of Orthopaedics and Traumatology Istanbul Kartal Dr. Lutfi Kirdar City Hospital Istanbul Turkey; ^5^ Department of Orthopaedics and Traumatology, Brandenburg Medical School Theodor Fontane University Hospital Brandenburg/Havel Brandenburg/Havel Germany; ^6^ Faculty of Health Sciences Brandenburg Brandenburg Medical School Theodor Fontane Brandenburg/Havel Germany

**Keywords:** anterior cruciate ligament (ACL) surgery, artificial intelligence (AI), ChatGPT, deep research, patient education

## Abstract

**Purpose:**

This study compares ChatGPT‐4o, equipped with its *deep research* feature, and DeepSeek R1, equipped with its *deepthink* feature—both enabling real‐time online data access—in generating responses to frequently asked questions (FAQs) about anterior cruciate ligament (ACL) surgery. The aim is to evaluate and compare their performance in terms of accuracy, clarity, completeness, consistency and readibility for evidence‐based patient education.

**Methods:**

A list of ten FAQs about ACL surgery was compiled after reviewing the Sports Medicine Fellowship Institution's webpages. These questions were posed to ChatGPT and DeepSeek in research‐enabled modes. Orthopaedic sports surgeons evaluated the responses for accuracy, clarity, completeness, and consistency using a 4‐point Likert scale. Inter‐rater reliability of the evaluations was assessed using intraclass correlation coefficients (ICCs). In addition, a readability analysis was conducted using the Flesch–Kincaid Grade Level (FKGL) and Flesch Reading Ease Score (FRES) metrics via an established online calculator to objectively measure textual complexity. Paired *t* tests were used to compare the mean scores of the two models for each criterion, with significance set at *p* < 0.05.

**Results:**

Both models demonstrated high accuracy (mean scores of 3.9/4) and consistency (4/4). Significant differences were observed in clarity and completeness: ChatGPT provided more comprehensive responses (mean completeness 4.0 vs. 3.2, *p* < 0.001), while DeepSeek's answers were clearer and more accessible to laypersons (mean clarity 3.9 vs. 3.0, *p* < 0.001). DeepSeek had lower FKGL (8.9 vs. 14.2, *p* < 0.001) and higher FRES (61.3 vs. 32.7, *p* < 0.001), indicating greater ease of reading for a general audience. ICC analysis indicated substantial inter‐rater agreement (composite ICC = 0.80).

**Conclusion:**

ChatGPT‐4o, leveraging its *deep research* feature, and DeepSeek R1, utilizing its *deepthink* feature, both deliver high‐quality, accurate information for ACL surgery patient education. While ChatGPT excels in comprehensiveness, DeepSeek outperforms in clarity and readability, suggesting that integrating the strengths of both models could optimize patient education outcomes.

**Level of Evidence:**

Level V.

**Abbreviations**
ACL: anterior cruciate ligament
ChatGPT: Chat Generative Pre‐Trained Transformer
FAQ: frequently asked question
FKGL: flesch–kincaid grade level
FRES: flesch reading ease score
ICC: intraclass correlation coefficient
LLM: large language model

## INTRODUCTION

Patients increasingly rely on online health information [3], yet the quality of orthopaedic resources, particularly for anterior cruciate ligament (ACL) surgery, remains inconsistent [26]. Misinformation and overly complex language, often exceeding the recommended grade reading level, hinder patient understanding and decision‐making [8]. There is a large potential within AI for patient education in terms of adequate complexity, comprehensibility and availability [25]. While large language models (LLMs) such as ChatGPT have shown potential to address gaps in providing reliable and easy‐to‐read data as well as providing assistance in research and writing [4, 5, 9, 14, 15, 21], early versions faced criticism for generating incomplete, outdated, or hallucinated content [15]. For instance, studies revealed that the earlier version of ChatGPT‐3.5 produced unsatisfactory answers to 60% of ACL surgery questions, with readability levels akin to college texts, and even fabricated citations in literature reviews [11]. The integration of research capabilities with real‐time online knowledge retrieval—alongside transparent reasoning features such as *deep research* (ChatGPT) and *deepthink* (DeepSeek), represents a recent advancement in AI chatbot technology introduced in early 2025. These features enable large language models to access up‐to‐date medical data, reduce hallucinations, and improve overall reliability [16]. Preliminary evaluations of OpenAI's Deep Research tool indicate that, while it generates clear and well‐referenced summaries with accurate surface‐level understanding, it demonstrates limitations in prioritizing recent findings, discerning nuanced concepts and capturing broader scientific context [20]. By providing easily available, tailored explanations of medical issues and treatment alternatives, AI chatbots have the ability to improve patient education. For orthopaedic surgeons, these tools could help simplify communication, cut time spent on recurrent counselling, and increase patient knowledge and satisfaction. Their inclusion into clinical practice could promote more knowledgeable decisions and improved patient involvement. Recent evaluations of ChatGPT's *deep research* mode demonstrated its ability to synthesize valid, influential literature, signalling a transformative shift in AI's clinical utility [12].

Emerging models like DeepSeek, a multimodal LLM launched in 2025, further expand this landscape [16]. Retrieval‐augmented generation, a cornerstone of *deep research* and *deepthink* frameworks, allows both models to integrate current guidelines and literature, addressing prior shortcomings in accuracy and consistency. Yet differences in training data and design philosophies may lead to divergent outcomes: ChatGPT's generative style might favour comprehensiveness at the cost of brevity, while DeepSeek's newer architecture could prioritize clarity and simplicity [16].

This study directly compares ChatGPT and DeepSeek, both equipped with deep research/think capabilities, in generating responses to ACL surgery frequently asked questions (FAQs). Accuracy, clarity, completeness and consistency, key metrics for patient education, are evaluated to determine how these models perform in evidence‐based information delivery. It is hypothesized that DeepSeek, as a newer model, will excel in clarity and consistency due to its streamlined design, while ChatGPT's established framework will yield more complete but potentially verbose answers.

## METHODS

A prospective, comparative evaluation of two AI chatbot models, ChatGPT (OpenAI) and DeepSeek (DeepSeek Inc.) was conducted to assess their performance in generating patient education content about ACL injuries and reconstruction surgery. The study focused on evaluating the quality of responses produced by each model when equipped with extended research capabilities. Ten questions were selected from the FAQ sections of commonly visited orthopaedic websites, focusing on those that were most frequently and consistently featured across multiple sources. Selection was further guided by input from two experienced sports medicine specialists to ensure clinical relevance. These questions addressed the spectrum of information needs for patients considering or undergoing ACL reconstruction, including injury mechanisms, symptoms, diagnostic approaches, treatment options, surgical procedures, rehabilitation protocols, risks, and expected outcomes (e.g., ‘How does an ACL injury occur?’ and ‘What is the recovery process after ACL surgery?’). All questions are listed in Table S1. Both chatbot models were prompted to provide informative responses to the questions from a patient's perspective.

## ARTIFICIAL INTELLIGENCE MODELS AND DEEP RESEARCH CONFIGURATION

### ChatGPT configuration

The study employed ChatGPT (GPT‐4o architecture) with its Deep Research feature activated. This configuration enabled ChatGPT to search external online sources, such as medical journals, guidelines, and reputable health websites, during response generation, bypassing reliance on its pre‐trained internal knowledge (last updated in 2021). After receiving a query, the ChatGPT interface performed real‐time web searches to retrieve and integrate relevant content. To align with patient education standards, ChatGPT was programmed to prioritize concise, accurate and complete explanations, including simplified definitions of medical terminology to enhance clarity. Explicit reference citations were omitted to simulate typical patient interactions, where AI‐generated responses paraphrase evidence‐based information without formal citations.

### DeepSeek configuration

DeepSeek Version R1 (released 2025) was utilized with its DeepThink feature, which similarly allowed access to external medical resources. Consistent with ChatGPT's instructions, DeepSeek was configured to generate responses tailored for patient education, emphasizing correctness and comprehensiveness. Queries were executed in isolated sessions to prevent cross‐model bias, ensuring no prior answers or feedback influenced subsequent responses.

### Evaluation criteria

Responses from ChatGPT and DeepSeek were systematically organized by question. Overt model identifiers were removed from all answers to ensure blinded evaluation. A panel of two orthopaedic surgeons specializing in sports medicine independently assessed each response using four criteria adapted from validated health information quality metrics [2, 27].

### Assessed properties

#### Accuracy (1–4 points)

Factual correctness was evaluated against standard references (e.g., orthopaedic textbooks, clinical guidelines). Scores ranged from 1 (*mostly incorrect/misleading*) to 4 (*fully accurate, no errors*).

#### Clarity (1–4 points)

Understandability for laypersons was assessed, focusing on language simplicity, avoidance of jargon, and logical flow. Scores ranged from 1 (*very confusing/technical*) to 4 (*clear, simple, well‐explained*).

#### Completeness (1–4 points)

Comprehensiveness in addressing all key aspects of the question (e.g., treatment options, rehabilitation, outcomes). Scores ranged from 1 (*omits most key points*) to 4 (*holistic, no significant omissions*).

#### Consistency (1–4 points)

Internal consistency (no self‐contradiction) and external consistency (alignment with medical consensus/guidelines) were evaluated. Scores ranged from 1 (*contradictory/non‐compliant*) to 4 (*fully consistent with clinical standards*).

### Readability

To assess the readability of AI‐generated patient education materials, a publicly accessible online readability calculator (https://www.readabilityformulas.com) was utilized [24]. This tool employs validated readability indices, including the Flesch–Kincaid Grade Level (FKGL) and the Flesch Reading Ease Score (FRES). These metrics are derived from computational analysis of linguistic features, specifically the average sentence length (measured as words per sentence) and syllabic complexity (quantified as syllables per word) [10, 17].

The full‐text responses from ChatGPT and DeepSeek were individually entered into the readability calculator for analysis. Mean FKGL and FRES were calculated by averaging results across all responses for each model. Lower FKGL (indicating simpler reading grade levels) and higher FRES values (suggesting easier comprehension) were interpreted as markers of greater accessibility, aligning with guidelines for low‐literacy audiences. This approach enabled consistent comparison of text complexity between AI systems using standardized metrics. Evaluation of resulting scores was based on the grading criteria defined in previous studies [10, 17].

FKGL:

- 0–5: Easily understood; suitable for primary school level
- 6–9: Suitable for middle to high school readers
- 10 and above: Advanced content, typically requiring college‐level literacy

FRES:

- 90–100: Very easy; appropriate for 11‐year‐olds
- 60–70: Standard readability; suitable for readers aged 13–15
- 30–50: Difficult; suitable for university‐level readers
- 0–30: Very difficult; comparable to academic or technical documents.

### Reliability and bias mitigation

Interrater reliability was assessed to verify the consistency of the scoring system and ensure evaluators shared a common interpretation of the criteria [19]. Intraclass correlation coefficients (ICCs) were calculated for each of the four criteria (accuracy, clarity, completeness and consistency). Intrarater reliability analysis was conducted using a one‐way mixed‐effects model, with each evaluator reassessing the same set of responses after a 1‐week interval without access to prior scores. ICC values were interpreted as follows: poor (<0.50), moderate (0.50–0.75), good (0.75–0.90) and excellent (>0.90) [18].

In line with recommendations for minimizing bias [23], evaluators were kept unaware of the AI model responsible for each response. Answers were anonymized, randomized and labelled with generic codes prior to evaluation. Evaluators were informed only that responses originated from AI systems, ensuring uniform scrutiny across all outputs. Evaluators had no prior involvement in answer generation and did not discuss their assessments with one another; discrepancies in scoring were reflected in reliability metrics, as reported in the results.

### Statistical analysis

Analyses were conducted using SPSS Statistics v28 (IBM Corp). Scores from the two evaluators were averaged per answer and criterion to generate a single composite score, resolving minor discrepancies (e.g., scores within one point of each other). These averaged scores were treated as paired data, as both models addressed identical queries.

Mean scores for ChatGPT and DeepSeek were compared using paired *t* tests for each criterion. Normality of score distributions was assessed via Shapiro–Wilk tests; however, due to the small sample size (10 question pairs), the Central Limit Theorem was cautiously invoked for parametric testing.

A composite quality metric (total score out of 16) was calculated by summing scores across all four criteria for each answer. Paired *t* tests compared total scores between models, though primary emphasis remained on individual criteria to avoid masking trade‐offs (e.g., clarity vs. completeness).

Statistical significance was set at *p* < 0.05 (two‐tailed). Results are reported as mean ± standard deviation (SD).

## RESULTS

### Interrater and intrarater reliability analysis

The interrater reliability analysis demonstrated substantial agreement between evaluators, with ICCs of 0.81 for accuracy, 0.79 for clarity, 0.76 for completeness and 0.85 for consistency. The composite ICC across all evaluation criteria was 0.80. These values suggest that the scoring criteria were applied consistently by different evaluators.

The resulting ICCs for intrarater reliability were 0.62 for the first evaluator and 0.67 for the second. These values indicate a moderate and repeatable scoring pattern, reinforcing the reliability of the assessment process over time.

### Assessed properties

Table 1 summarizes the mean scores across all 10 questions. On the 4‐point scale, both ChatGPT and DeepSeek, with deep research/deepthink features, showed high accuracy (3.9 ± 0.3 vs. 3.9 ± 0.5, *p* = *n.s*) and consistency (4.0 ± 0.0 vs. 4.0 ± 0.0, *p* = *n.s*). However, ChatGPT scored lower in clarity (3.0 ± 0.5 vs. 3.9 ± 0.3, *p* < 0.001) but higher in completeness (4.0 ± 0.00 vs. 3.2 ± 0.42, *p* < 0.001), highlighting a trade‐off between completeness and clarity.

**Table 1:** Mean evaluation and readability scores (±SD) for ChatGPT and DeepSeek across ten ACL surgery questions.

Criterion	ChatGPT 4o (Deep Research enabled)	DeepSeek R1 (DeepThink enabled)	*p* value
Accuracy (1–4)	3.9 ± 0.3	3.9 ± 0.5	0.99 (n.s.)
Clarity (1–4)	3.0 ± 0.5	3.9 ± 0.3	<0.001**
Completeness (1–4)	4.0 ± 0.0	3.2 ± 0.4	<0.001**
Consistency (1–4)	4.0 ± 0.0	4.0 ± 0.0	1.00 (n.s.)
FKGL	14.2 ± 1.3	8.9 ± 1.0	<0.001**
FRES	32.7 ± 5.1	61.3 ± 6.0	<0.001**

Abbreviations: ACL, anterior cruciate ligament; FKGL, Flesch–Kincaid Grade Level; FRES, Flesch Reading Ease Score; n.s., not significant; SD, standard deviation.

** statistically significant.

### Readability

Readability analysis revealed significant differences between the responses generated by ChatGPT and DeepSeek (Table 1), with ChatGPT requiring a significantly higher reading level (*p* < 0.001, paired *t* test). ChatGPT responses had an FKGL of 14.2 ± 1.3 (advanced content, typically requiring college‐level literacy) and a FRES of 32.7 ± 5.1 (difficult; suitable for university‐level readers), whereas DeepSeek responses had an FKGL of 8.9 ± 1.0 (suitable for middle to high school readers) and a FRES of 61.3 ± 6.0 (standard readability; suitable for readers aged 13–15).

## DISCUSSION

The most critical finding of this study is the distinct trade‐off between completeness and clarity in AI‐generated patient education materials for ACL surgery. Utilizing a deep research feature, with the ability to retrieve current medical information, ChatGPT produced significantly more comprehensive responses, but at the expense of reduced readability. Conversely, DeepSeek prioritized accessibility, aligning with recommended health literacy standards. This divergence underscores the challenge of balancing detail and simplicity in patient‐facing AI tools.

The present study found that the incorporation of real‐time retrieval functionality significantly enhanced the accuracy and consistency of AI‐generated content. Previous investigations identified shortcomings in earlier ChatGPT versions, reporting an accuracy rate of only 65% for ACL surgery‐related queries [14] and highlighting instances where ChatGPT‐3.5 offered outdated treatment recommendations and failed to reflect current medical guidelines [13]. By equipping both ChatGPT‐4 and DeepSeek with real‐time retrieval capabilities, the current study addressed these earlier limitations, particularly the issues of outdated and hallucinated information [13, 15].

Importantly, none of the responses generated by either ChatGPT‐4 or DeepSeek using the deep research feature were deemed factually incorrect by expert evaluators. This improvement is consistent with findings in other medical domains, such as diabetes education, where retrieval‐augmented models demonstrated increased accuracy [28]. Notably, this study is among the first to systematically evaluate AI‐generated content produced by chatbots equipped with real‐time research functionality. The findings indicate that both models effectively leveraged their retrieval capabilities to access up‐to‐date and credible sources, thereby ensuring that their responses aligned with current standards of care. Additionally, both models exhibited a high degree of consistency with established medical consensus, suggesting that access to reliable external sources enables AI models to produce not only internally coherent responses but also content that aligns with evidence‐based clinical guidelines. These findings stand in contrast to earlier reports involving previous versions of ChatGPT‐3.5 and 4, which demonstrated variability and frequent departures from expert consensus [1].

Despite the strengths in accuracy and consistency, a notable divergence was observed between the models in terms of clarity and completeness. ChatGPT consistently generated more comprehensive responses, covering a broader range of information, including psychological aspects of recovery and alternative treatments like bracing or physical therapy. However, this breadth came at the expense of clarity, with ChatGPT scoring significantly lower than DeepSeek in clarity evaluations (3.0 vs. 3.9, *p* < 0.001). Moreover, ChatGPT's responses were more complex, requiring a higher reading level for comprehension (FKGL = 14.2; FRES = 32.7), indicating significant difficulty. These findings align with previous critiques of GPT‐4's patient communication style [8] and echo earlier studies showing ChatGPT responses exceed the average American reading levels by more than nine grade levels [8]. However, some literature emphasizes the importance of providing more comprehensive explanations for patient understanding [29]. In contrast, DeepSeek's outputs demonstrated superior readability, more closely aligning with the *American Medical Association* (AMA) and Centers for Disease Control and Prevention (CDC) recommendations that patient education materials be written at a sixth‐ to eighth‐grade level [7]. DeepSeek's streamlined responses, adhering more closely to these guidelines, support earlier findings that simpler content improves patient recall [6]. The observed trade‐off between comprehensive and concise outputs mirrors a common clinical challenge: excessive detail may overwhelm patients, while oversimplification risks omitting critical information [22].

Importantly, while AI tools such as ChatGPT and DeepSeek can standardize and enhance patient education, they are not substitutes for professional medical judgement in regard to ACL injury or reconstruction. Each ACL injury is unique and must be evaluated individually by an orthopaedic surgeon, considering factors such as associated injuries (e.g., medial collateral ligament, meniscus and cartilage damage), patient activity level, and overall health. The questions posed to the AI in this study were intentionally general; in clinical practice, treatment decisions often vary significantly based on individual patient characteristics. Moreover, there is a potential risk that patients may overly rely on AI‐generated content to guide their expectations around treatment and surgical outcomes, which underscores the importance of direct physician–patient communication. AI‐generated content should therefore be used to supplement, not replace, personalized clinical evaluation and discussion [14, 15, 16]. This study underscores the potential role of AI chatbots as a supplementary tool to improve patient education in orthopaedics, highlighting their value in enhancing understanding while reinforcing the necessity of individualized clinical care.

This study is not without limitations. The sample consisted of only ten questions, selected to reflect common patient inquiries, but was insufficient to capture the full variability in model performance. These questions were general in nature—focusing on topics such as injury mechanisms and rehabilitation—and did not evaluate the AI's ability to address more complex clinical scenarios, which may present additional challenges in clarity and accuracy. Additionally, evaluations were conducted by medical professionals rather than patients, which may limit the generalizability of readability and clarity assessments. Future research should incorporate diverse patient populations and directly assess comprehension, satisfaction and clinical utility.

## CONCLUSION

ChatGPT‐4o and DeepSeek R1, both equipped with deep research capabilities, deliver accurate and consistent educational content on ACL surgery but differ significantly in communication style. ChatGPT offers more comprehensive and detailed responses, whereas DeepSeek focuses on clarity and brevity. By integrating the complementary strengths of these models—through hybrid systems or customized prompting—the effectiveness of AI‐assisted patient education in orthopaedics could be enhanced. As adjunct tools, advanced AI chatbots show considerable potential in improving patient understanding and fostering greater engagement in patient care.

## AUTHOR CONTRIBUTIONS

All listed authors have contributed substantially to this work: All authors have read and approved the final manuscript to be submitted and published. Conceptualization: Onur Gültekin, Baris Yilmaz, Mehmet Halis Cerci, Bekir Eray Kilinc, Hüsnü Yilmaz and Mahmut Enes Kayaalp. Methodology: Onur Gültekin, Baris Yilmaz, Mehmet Halis Cerci, Hüsnü Yilmaz, Robert Prill and Mahmut Enes Kayaalp. Formal analysis and investigation: Onur Gültekin, Jumpei Inoue and Bekir Eray Kilinc. Writing—original draft preparation: Onur Gültekin, Jumpei Inoue, Hüsnü Yilmaz, Robert Prill and Mahmut Enes Kayaalp. Writing—review and editing: Onur Gültekin, Jumpei Inoue, Robert Prill and Mahmut Enes Kayaalp. Funding acquisition: Not applicable. Resources: Jumpei Inoue, Hüsnü Yilmaz, Robert Prill and Mahmut Enes Kayaalp. Supervision: Baris Yilmaz, Bekir Eray Kilinc and Mahmut Enes Kayaalp.

## CONFLICT OF INTEREST STATEMENT

Robert Prill: Associate editor of KSSTA. Mahmut Enes Kayaalp: Associate editor of KSSTA, member of U‐45 Committee, ESSKA. The remaining authors declare no conflicts of interest.

## ETHICS STATEMENT

The ethics statement is not available.

## Supporting information

Supporting information.

## Data Availability

Available upon request.
